# Intensive care referral and admission: do the criteria for liver disease match?

**DOI:** 10.1186/cc14464

**Published:** 2015-03-16

**Authors:** J McPeake, CR Soulsby, T Quasim, J Kinsella

**Affiliations:** 1 University of Glasgow, UK

## Introduction

Hospital admission and mortality rates for patients with cirrhosis in the UK are rising [1]. Cirrhotic patients are physiologically challenged and at increased risk of sepsis and death [2]. Mortality rates for cirrhosis in nontransplant ICUs are up to 37% [3]. Increased availability of medical therapies and public expectation places pressure on limited intensive care resources. There is a lack of research into factors used to decide which patients to admit or refer to the ICU.

## Methods

A prospective survey was sent to all consultant gastroenterologists and consultant intensivists in Scotland. Each recipient rated the significance of 18 physiological and social criteria on their decision to refer or admit a patient to intensive care from 1 to 5, with 1 being no influence and 5 denoting significant impact on the decision. Recipients listed additional criteria used in their own practice and asked whether they would admit or refer individual grades of Child-Pugh cirrhosis with either a gastrointestinal bleed or sepsis.

## Results

Thirty-five consultant gastroenterologists and 65 intensive care consultants responded, representing a response rate of 34% and 45% respectively. The only criterion given an average rating of 5 by both gastroenterologists and intensivists was Child-Pugh score when stable. Presence on the transplant list, referral secondary to bleeding varices, recent discharge from the ICU, abstinence from alcohol, nutritional status, age under 30 and more than one additional organ failure all scored 4 or 5 from both groups. Sex, employment, smoking or drug use, deprivation and positive virology status did not influence the decision to refer or admit patients. Clinicians reported compliance with medication and outpatient appointments plus an obvious precipitant factor as important features in their decision. The majority of respondents would refer or admit all grades of Child-Pugh cirrhosis with gastrointestinal bleeding. Most would refer or admit Child-Pugh A or B with sepsis. A total 76.5% of gastroenterologists would refer Child-Pugh C cirrhosis with sepsis but only 33.3% of intensivists would accept.

## Conclusion

Referral and admission decisions for patients with cirrhosis are multifactorial. Child-Pugh status when stable appears to be of greatest significance. The difference in opinion of admission of patients with Child-Pugh C with sepsis requires further evaluation.
